# Biological noise and H2A.Z: a promising connection for language

**DOI:** 10.3389/fgene.2014.00463

**Published:** 2015-01-09

**Authors:** Antonio Benítez-Burraco

**Affiliations:** Spanish Philology and its Didactics, University of HuelvaHuelva, Spain

**Keywords:** biological noise, canalization, language evolution, anatomically-modern humans, Neanderthals, H2A.Z, RUNX2, SIRT1

In their paper “How does evolution tune biological noise?” (*Front. Genet*. *5:374*) Richard and Yvert (2014) argue that biological noise (a source of variation regarding phenotypic traits) is under genetic control and consequently, is an evolvable trait. The authors focus on *H2A.Z* to illustrate their hypothesis and claim that this gene may act as a buffering device for this kind of variation. The examples provided by them range from *S. cerevisae* to *A. thaliana*, but we think that considerations from the fields of language disorders and language evolution may be adduced to lend credence to their hypothesis. This is the main goal of this commentary. Additionally, we will present evidence showing that *H2A.Z* is a gene of interest regarding the genetic underpinnings of the human faculty for language.

In a recent paper (Benítez-Burraco and Boeckx, 2014) we have claimed that, contrary to what has usually been assumed by linguists, the human faculty for language is not a homogeneous biological entity. Specifically, we showed that variation pervades language at all levels of analysis, from the neurobiological to the genetic layer. We further argued that this variation is canalized, to the extent that a functional language faculty almost invariably emerges in all subjects at the term of growth. What is more, environmental and endogenous perturbations (for example, in the form of mutations in genes related to language) bring about a limited set of deviant phenotypes only (i.e., language disorders), which can be conceived as decanalized states resulting from the uncovering of cryptic variation (following Gibson, 2009). In our paper we further claimed that, because of its biological novelty, the emergence of the language faculty can be conceived as a set of changes that decanalized the primate cognome, pushing it away from the robust equilibrium achieved after millions of stabilizing selection. Consequently, one would expect that some buffering mechanism have been reinforced in modern humans (henceforth AMHs) in order to recanalize all this new variation and make the faculty of language as robust as it is in current human populations.

In parallel to this line of argument, we have put forth in a recent series of papers (Boeckx and Benítez-Burraco, 2014a,b) a gene network that we think is implicated in the development of the language-ready brain, that is, our species-specific brain properties that enable us to acquire and use languages (see Arbib, 2012 for details). According to our view this language-readiness resulted from a rewiring of the connections between sub-cortical (specifically, the thalamus) and cortical structures, a rewiring that is also manifested in a new developmental trajectory of skull formation, which gives rise to the characteristic globular head shape of AMHs, resulting from a relative expansion of the parietal and cerebellar regions compared to extant primates and extinct hominins (Neubauer et al., 2010). Our set of genes is centered on *RUNX2*, which controls different aspects of skull and brain development (Stein et al., 2004; Reale et al., 2013) and whose promoter region shows two derived alleles in AMHs (Perdomo-Sabogal et al., 2014).

It is with this background in mind that we have approached Richard and Yvert's (2014) take on *H2A.Z*. One of the genes that control H2A.Z levels is *SIRT1*. SIRT1 reduces H2A.Z levels via proteasome-mediated degradation (Baptista et al., 2013); not surprisingly given its role in the control of noise, as discussed by Richard and Yvert, high levels of H2A.Z (and low levels of SIRT1) are observed in different cancers (Baptista et al., 2013 and references herein). *SIRT1* was known to be involved in cell survival, differentiation, and metabolism, but ample evidence now suggests that in the brain SIRT1 regulates different neural processes via deacetylation of histones and other proteins (like AKT1), including axon formation and elongation (Li et al., 2013), neural precursor activity and differentiation (e.g., high levels of SIRT1 signaling prevents neurogenesis and neural differentiation) (Saharan et al., 2013), and memory formation (Gao et al., 2010). Interestingly, *H2A.Z* has been recently linked to cognitive function too, as a direct regulator of memory consolidation systems (Zovkic et al., 2014). At the same time, a functional link also exists between SIRT1 and RUNX2. During osteogenesis SIRT1 increases the mRNA levels of *RUNX2* and deacetylates RUNX2, ultimately promoting osteoblast differentiation (Shakibaei et al., 2012; Srivastava et al., 2012). The higher level of RUNX2 in the brain, the shorter interval time in which skull sutures remain open (Stein et al., 2004). Overall, a functional links seems to exist between *SIRT1*, *RUNX2*, and *H2A.Z*.

In our 2014a piece, we hypothesized that lower levels of RUNX2 in AMHs compared to Neanderthals (plausibly resulting from the changes in *RUNX2* promoter) may explain some of the differences between the AMH and the Neanderthal skulls, brains, and cognitive abilities. Importantly, lower levels of RUNX2 are expected to correlate with lower levels of SIRT1 and with higher levels of H2A.Z at the brain level. If Richard and Yvert (2014) are right, this circumstance may have helped to buffer the molecular noise resulting from the changes occurred in the RUNX2 network that brought about a language-ready brain (and ultimately, from the decanalization of the primate cognome). But because of the involvement of both *SIRT1* and *H2A.Z* in memory consolidation, we should expect that it contributed as well to the remodeling of the memory systems that is characteristic of our species, in particular, to the enhancement of the working memory and to a faster transition from declarative to procedural performance, a process important for language evolution and to which *FOXP2*, the famous language gene, also contributes to Schreiweis et al. (2014). Further evidence confirms the relevance of both genes for the emergence of modern language. Hence, *SIRT1* is functionally related to some other genes encompassing the network we think important for the language-readiness, including *TP53*, *EP300*, *HES1*, and *CLOCK* (see Boeckx and Benítez-Burraco, 2014a,b for details). Similarly, according to String 9.1 [a tool that predicts direct/physical and indirect/functional associations between proteins based on genomic context, high-throughput experiments, conserved coexpression, and text mining (Szklarczyk et al., 2011)], some of the genes that have changed in AMHs compared to Neanderthals/Denisovans and that are part of our network are co-expressed along with *H2A.Z*, namely *SPAG5* and *ANAPC10* (see Benítez-Burraco and Boeckx, submitted for details).

In sum, we regard the hypothesis by Richard and Yvert of outstanding interest concerning language evolution and the biological nature of the human faculty for language. Moreover, we think that the genetic aspects highlighted here, if explored experimentally, will allow us to gain a better view of the genetic underpinnings of language and the molecular mechanisms that channel variation at all levels of analysis.

## Conflict of Interest Statement

The author declares that the research was conducted in the absence of any commercial or financial relationships that could be construed as a potential conflict of interest.
